# Nitroglycerin use and adverse clinical outcomes in elderly patients with acute coronary syndrome

**DOI:** 10.1136/openhrt-2023-002494

**Published:** 2024-01-12

**Authors:** Soichi Komaki, Yunosuke Matsuura, Hiroki Tanaka, Kohei Moribayashi, Yoshimasa Yamamura, Kazumasa Kurogi, Takeshi Ideguchi, Nobuyasu Yamamoto, Michikazu Nakai, Toshihiro Tsuruda, Koichi Kaikita

**Affiliations:** 1 Division of Cardiovascular Medicine and Nephrology, Department of Internal Medicine, Faculty of Medicine, University of Miyazaki, Miyazaki, Japan; 2 Department of Cardiovascular Medicine, Miyazaki Prefectural Nobeoka Hospital, Nobeoka, Miyazaki, Japan; 3 Clinical Research Support Center, University of Miyazaki Hospital, Miyazaki, Japan; 4 Department of Hemo-Vascular Advanced Medicine, Faculty of Medicine, University of Miyazaki, Miyazaki, Japan

**Keywords:** Acute Coronary Syndrome, Outcome Assessment, Health Care, Pharmacology, Clinical, Percutaneous Coronary Intervention

## Abstract

**Objective:**

The primary care for acute coronary syndrome (ACS) includes the administration of nitroglycerin (GTN). This study aimed to investigate the association between the use of GTN before percutaneous coronary intervention (PCI) for ACS and clinical outcomes.

**Methods:**

Nine-hundred and forty-seven patients who underwent PCI for ACS were examined and classified into two groups: those who were treated with GTN before PCI (GTN group) and those who were not (non-GTN group). The incidence of major adverse cardiovascular events (MACE), which consist of all-cause mortality, non-fatal myocardial infarction, stroke and rehospitalisation for heart failure at 1 year, was compared between the two groups.

**Results:**

This study identified 289 patients with ACS who used GTN preceding PCI. Pre-PCI systolic blood pressure was significantly lower in the GTN group than in the non-GTN group (median (IQR); 132.0 (110.0–143.5) mm Hg vs 134.0 (112.0–157.0) mm Hg, respectively, p=0.03). Multivariate Cox regression analysis indicated that GTN use preceding PCI showed an independent association with the incidence of MACE (HR 1.57; 95% CI 1.09–2.28; p=0.016). Overall, the incidence of MACE 1 year after PCI for ACS was significantly higher in the GTN group than in the non-GTN group (log-rank test, p=0.024); however, this trend was consistently found in elderly patients aged ≥75 years (p=0.002) but not in non-elderly patients aged <75 years (p=0.773).

**Conclusions:**

GTN use preceding PCI for ACS is associated with lower blood pressure and adverse clinical outcomes in elderly patients.

**WHAT IS ALREADY KNOWN ON THIS TOPIC**

- The primary care for acute coronary syndrome (ACS) includes the administration of nitroglycerin (GTN).
- Whether GTN use preceding primary percutaneous coronary intervention (PCI) is associated with major adverse cardiovascular events (MACE) remains unknown in the era of the changes in patient characteristics and advances in ACS management, including PCI and optimal medical therapy.

**WHAT THIS STUDY ADDS**

- GTN use preceding primary PCI for ACS showed an independent association with the incidence of the 1-year MACE.
- In particular, the GTN group had a significantly higher incidence of MACE than the non-GTN group in elderly patients (age ≥75 years).

**HOW THIS STUDY MIGHT AFFECT RESEARCH, PRACTICE OR POLICY**

- GTN use preceding primary PCI was associated with adverse clinical outcomes in elderly patients with ACS.
- In the optimisation of primary management for patients with ACS, the pros and cons of GTN use might need to be debated and examined separately for elderly and non-elderly patients.

## Introduction

Primary percutaneous coronary intervention (PCI)^1–3^ and optimal medical therapy (OMT), including antithrombotic therapy^4^ and lipid-lowering therapy,^5^ have significantly improved cardiovascular outcomes in patients with acute coronary syndrome (ACS). However, ACS remains a leading cause of death worldwide.^6–8^ To further improve clinical outcomes, strategies have been implemented to shorten the time from the onset of ACS to the initiation of the diagnostic process or medical intervention, which extends to the prehospital or pretransport phase.^9 10^ These management protocols, before primary PCI, include the administration of nitroglycerin (GTN).

GTN has long been incorporated as a first-line drug for ACS.^6–8^ This is primarily because it decreases venous return, thereby reducing left ventricular filling pressure and blood pressure^11^ and, consequently, myocardial oxygen demand.^12^ However, limited studies^13 14^ support the use of GTN for improving ACS outcomes; moreover, these investigations were mainly conducted over three decades ago when the clinical demographics of patients with ACS significantly differed from the current situation. The changes in patient characteristics (eg, an increased number of elderly patients) and advances in ACS management, including PCI and OMT, have raised the need to reassess the efficacy of GTN for clinical outcomes after primary PCI for ACS. Therefore, this study investigated the association between pre-PCI use of GTN and clinical outcomes in patients with ACS.

## Methods

### Study design and population

**Figure 1:**
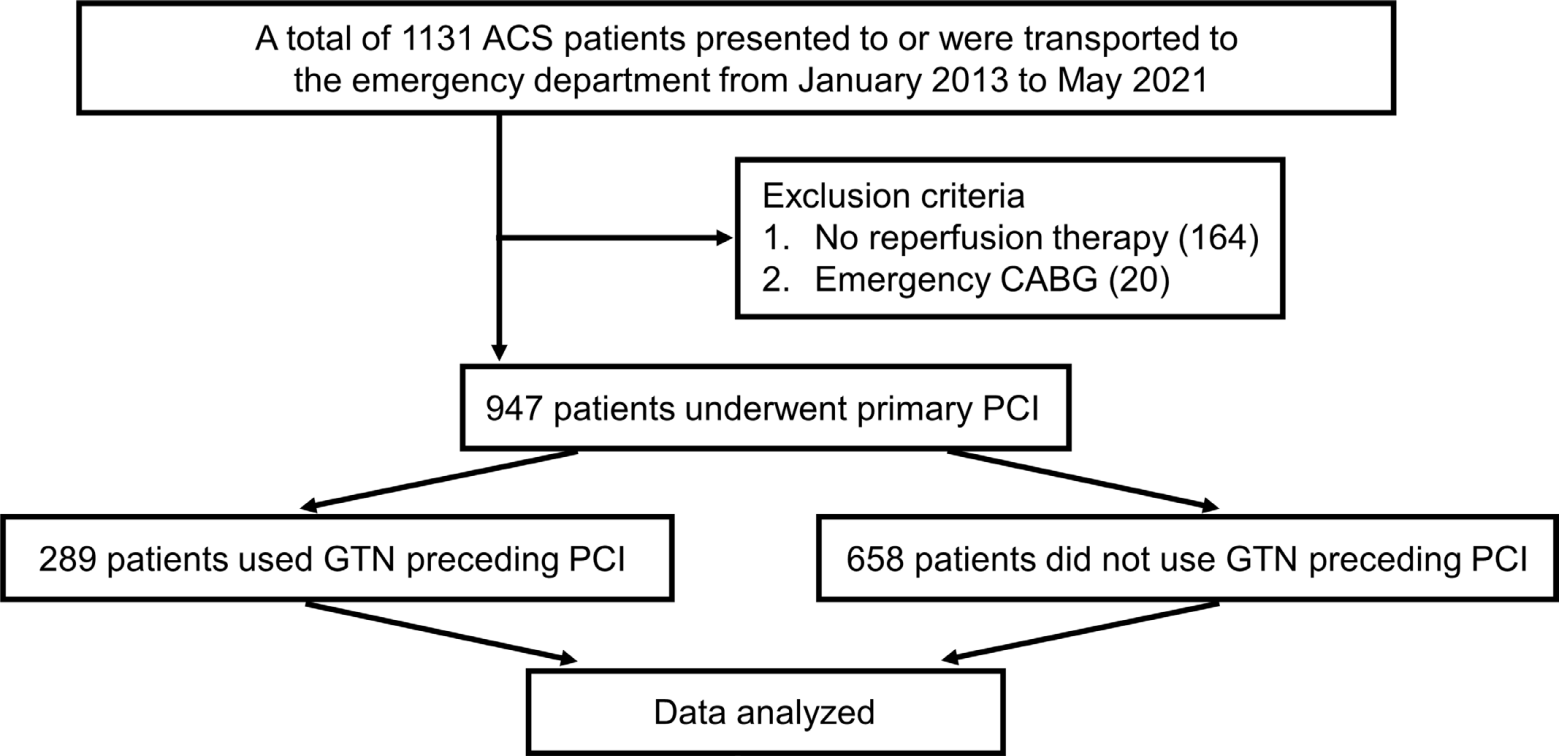
Flow chart of patient inclusion and exclusion. ACS, acute coronary syndrome; CABG, coronary artery bypass grafting; GTN, nitroglycerin; PCI, percutaneous coronary intervention.

We conducted a single-centre, retrospective, observational study of consecutive patients with ACS admitted to Miyazaki Prefectural Nobeoka Hospital from January 2013 to May 2021. Initially, 1131 patients were enrolled. Subsequently, patients who did not undergo revascularisation (n=164) and patients who underwent emergency coronary artery bypass surgery (n=20) were excluded, resulting in a total of 947 patients for the final analysis (figure 1, online supplemental methods S1). The use of GTN preceding primary PCI was defined as the administration of GTN in primary care before or after arrival at the emergency department (ED), except for GTN administration in the cardiac catheterisation laboratory. A review of medical records on drug use before and after arrival at our ED confirmed the following: (1) 289 patients received sublingual, oral spray or intravenous administration of GTN before PCI (250 patients before transport to our institute and 39 patients due to immediate care after arrival at the ED); (2) 144 patients received a loading dose of antiplatelet agents at the prior hospital and (3) 45 patients received unfractionated heparin at the prior hospital. In this study, patients with ACS who underwent PCI were divided into the GTN group and the non-GTN group according to the use of GTN before PCI (online supplemental methods S2). The association between the use of GTN before PCI and clinical outcomes was examined. The need for obtaining informed consent was waived owing to the low-risk nature of the study. This study was disclosed and announced on the hospital website (https://nobeoka-kenbyo.jp/sinryoka/junkankika/) in case any patient wished to opt-out.

10.1136/openhrt-2023-002494.supp1 Supplementary data

### PCI procedures and pre-PCI and post-PCI medical management

All patients received aspirin and a P2Y12 inhibitor (clopidogrel or prasugrel) in accordance with ACS treatment guidelines.^6^ Specifically, a P2Y12 inhibitor was administered after the decision to perform emergency PCI based on coronary angiography results. Also, weight-adjusted intravenous heparin was administered before primary PCI (online supplemental methods S3). Coronary angiography and subsequent PCI were performed according to standard practice, and the culprit lesions requiring urgent revascularisation were determined by angiography in conjunction with electrocardiographic and echocardiographic findings. The door-to-balloon (D2B) time was measured from admission to achieving coronary reperfusion, and the Thrombolysis in Myocardial Infarction (TIMI) flow grades were evaluated before and after PCI. Successful reperfusion was defined as the confirmation of TIMI 3 flow on angiography. The indications for emergency coronary artery bypass grafting (CABG) included the following: (1) the presence of active ischaemia with contraindications for PCI; (2) successful PCI of the culprit lesion and further indication for CABG and (3) incomplete, insufficient or failed PCI. Patients with haemodynamic compromise refractory to medical treatment and revascularisation for ACS were treated with cardiac and respiratory assist devices, including intra-aortic balloon pumping, venoarterial extracorporeal membrane oxygenation, temporary pacing, non-invasive positive pressure ventilation and mechanical ventilation with endotracheal intubation, as appropriate. All patients undergoing primary PCI for ACS were treated in the coronary care unit. In detail, OMT was timely initiated, and cardiac monitoring, including peak creatine kinase (CK) assessment, electrocardiography and echocardiography, was performed. Additionally, a cardiac rehabilitation programme was appropriately provided as soon as possible after risk assessment for the development of acute complications after ACS in accordance with cardiac rehabilitation guidelines.^15^

### Clinical outcomes

Clinical follow-up information was obtained from medical records and/or telephone interviews with patients or their families. The primary outcome was the incidence of major adverse cardiovascular events (MACE) within 1 year, defined as a composite of all-cause death, non-fatal myocardial infarction (MI), stroke and rehospitalisation for heart failure. The secondary outcome was stroke and target lesion revascularisation (TLR) after stent implantation at the 1-year follow-up. TLR was defined according to the Academic Research Consortium criteria.^16^

### Statistical analysis

Categorical and continuous variables are presented as numbers with percentages and as median values with IQRs, respectively. The χ^2^ test or Fisher’s exact test was used for categorical variables, and the Mann-Whitney U test was used for continuous variables. Spearman’s rank test was used for scale variables. In the GTN and non-GTN groups, clinical variables such as patient characteristics (including comorbidities, history, laboratory data, medication and PCI procedure relevant factors) and clinical outcomes were compared. The cumulative incidence rates of MACE were calculated by the Kaplan-Meier method, with comparisons between the GTN and non-GTN groups being performed using log-rank tests. Univariate and multivariate Cox regression analyses were used to calculate HRs and corresponding 95% CIs for the incidence of MACE. Multivariate analyses were performed using statistically significant clinical variables from the univariate analysis. In addition, inverse probability weighting (IPW) was performed as a sensitivity analysis using the same covariates with multivariate analysis. The balance between the groups before and after matching was assessed using standardised differences (<0.1 was considered as well balanced). Statistical significance was defined as a p<0.05, and all statistical analyses were performed by using SPSS V.20.0 (IBM) and STATA V.18.

## Results

### Clinical characteristics in the GTN and the non-GTN groups

This study analysed 947 patients with a median age of 71 years and with 71.5% of male patients. Hypertension was the most common coronary risk factor, followed by dyslipidaemia, smoking and diabetes. Two hundred and eighty-nine patients (30.5%) received GTN preceding PCI as primary care for ACS in prehospital and in-hospital phases: 228 with sublingual or oral spray, 57 with intravenous and 4 with both. In the pre-PCI baseline characteristics, the GTN group (vs non-GTN group) had significantly lower systolic blood pressure (132 (110–143) mm Hg vs 134 (112–157) mm Hg, p=0.03), a trend towards lower diastolic blood pressure (80 (66–93) mm Hg vs 83 (69–94) mm Hg, p=0.08), and significantly higher prevalence of hypertension (82.6% vs 72.6%, p<0.001) and dyslipidaemia (76.8% vs 66.1%, p<0.001). In addition, the GTN group had a trend towards a higher proportion of patients who previously received PCI (17.9% vs 13.3%, p=0.065) (online supplemental table S1). In a survey of antithrombotic pharmacotherapy regimens before the onset of ACS, the GTN group showed significantly higher rates of aspirin (20.7% vs 12.7%, p=0.003) and warfarin use (2.4% vs 0.5%, p=0.016) and a trend towards higher rates of direct oral anticoagulant (DOAC) use (2.4% vs 0.8%, p=0.066), compared with the non-GTN group (online supplemental table S1).

No significant differences were found between the GTN and non-GTN groups regarding PCI approach site, target vessel, Killip classification, frequency of drug-eluting stent and bare metal stent use, peak level of CK, D2B time and frequency of imaging device use, except for that the GTN group showed a significantly lower proportion of right coronary artery (RCA) related infarction and temporary pacing therapy than the non-GTN group in emergency PCI procedures (online supplemental table S2). Regarding antithrombotic drugs at discharge, 98.9% of the patients received aspirin, 38.9% received clopidogrel, 59.6% received prasugrel, 2.3% received warfarin and 6.2% received DOACs. No difference in the proportion of antithrombotic drugs was observed between the GTN and non-GTN groups (online supplemental table S2).

### Clinical outcomes in the GTN and non-GTN groups

PCI was successfully performed for 942 (99.4%) patients. During the 1-year follow-up, the incidence of MACE was found in 146 (15.4%) patients: 88 (9.2%) with all-cause death, 13 (1.3%) with MI, 23 (2.4%) with stroke and 27 (2.8%) with heart failure requiring rehospitalisation. Compared with the non-GTN group, the GTN group exhibited a significantly higher incidence of MACE (19.3% vs 13.6%, p=0.025). In addition, a trend towards the higher incidence of TLR (7.2% vs 4.5%, p=0.089) was observed in the GTN than in the non-GTN group (table 1). In the GTN group, patients with MACE had significantly lower levels of systolic (130.0 (95.0–141.0) vs 132.0 (113.0–146.0) mm Hg, p=0.011) and diastolic blood pressure (76.5 (57.7–91.2) vs 84.0 (70.0–95.0) mm Hg, p=0.011) than those without MACE (online supplemental table S3).

**Table 1:** Clinical characteristics and the use of GTN before PCI in patients with ACS

	Total (n=947)	GTN group (n=289)	Non-GTN group (n=658)	P value
Age (years)	71.0 (63.0–80.0)	70.0 (62.0–80.0)	71.0 (63.0–80.0)	0.377
Elderly (≥75 years), n (%)	378 (39.9)	112 (38.7)	266 (40.4)	0.629
Male, n (%)	678 (71.5)	215 (74.3)	463 (70.3)	0.205
BMI (kg/m^2^)	23.4 (21.4–26.0)	23.7 (21.4–26.3)	23.4 (21.4–25.8)	0.114
Waist circumference (cm)	87.0 (82.0–93.0)	87.6 (82.0–94.5)	87.0 (81.0–93.0)	0.102
Systolic blood pressure (mm Hg)	132.0 (112.0–154.0)	132.0 (110.0–143.5)	134.0 (112.0–157.0)	**0.030**
Diastolic blood pressure (mm Hg)	81.0 (68.0–93.2)	80.0 (66.0–93.0)	83.0 (69.0–94.0)	0.081
Previous/current smoking, n (%)	465 (49.1)	139 (48.0)	326 (49.5)	0.682
Hypertension, n (%)	717 (75.7)	239 (82.6)	478 (72.6)	**<0.001**
Dyslipidaemia, n (%)	657 (69.3)	222 (76.8)	435 (66.1)	**<0.001**
Diabetes mellitus, n (%)	300 (31.6)	100 (34.6)	200 (30.3)	0.200
Metabolic syndrome, n (%)	330 (34.8)	97 (33.5)	233 (35.4)	0.348
Haemodialysis, n (%)	29 (3.0)	10 (3.4)	19 (2.8)	0.638
TG (mg/L)	106.0 (73.0–159.0)	104.0 (73.0–149.0)	106.0 (73.0–164.5)	0.410
HDL-C (mg/L)	48.0 (40.7–58.0)	48.0 (41.0–59.0)	48.0 (40.0–57.0)	0.211
LDL-C (mg/L)	121.0 (98.0–154.0)	123.0 (97.0–151.0)	121.0 (98.0–148.0)	0.604
HbA1c (%)	5.90 (5.50–6.50)	6.00 (5.60–6.60)	5.90 (5.50–6.40)	0.064
eGFR (mL/min per 1.73 m2)	61.4 (48.4–72.8)	62.0 (47.6–73.2)	61.2 (48.5–72.6)	0.932
LVEF (%)	56.8 (49.0–62.3)	56.1 (50.0–62.4)	56.8 (48.8–62.3)	0.924
STEMI, n (%)	748 (78.9)	226 (78.2)	522 (76.2)	0.694
MACE, n (%)	146 (15.4)	56 (19.3)	90 (13.6)	**0.025**
All-cause death, n (%)	88 (9.2)	34 (11.7)	54 (8.2)	0.082
MI, n (%)	13 (1.3)	4 (1.3)	9 (1.3)	0.984
Stroke, n (%)	23 (2.4)	9 (3.1)	14 (2.1)	0.364
Heart failure, n (%)	27 (2.8)	11 (3.8)	16 (2.4)	0.242
TLR, n (%)	51 (5.3)	21 (7.2)	30 (4.5)	0.089

Data are presented as medians (IQRs) or numbers (percentages). Bold values indicate statistical significance at the p <0.05 level.

ACS, acute coronary syndrome; BMI, body mass index; eGFR, estimated glomerular filtration rate; GTN, nitroglycerin; HbA1c, hemoglobin A1c ; HDL-C, high-density lipoprotein cholesterol; LDL-C, low-density lipoprotein cholesterol; LVEF, left ventricular ejection fraction; MACE, major adverse cardiovascular event; MI, myocardial infarction; PCI, percutaneous coronary intervention; STEMI, ST-elevation myocardial infarction; TG, triglyceride; TLR, target lesion revascularisation.

Table 2 shows the results of univariate and multivariate analyses for clinical variables associated with MACE that occurred during follow-up in all patients. Univariate analysis indicated that age, dyslipidaemia, chronic kidney disease, left ventricular ejection fraction (LVEF), Killip classification and the use of GTN were significantly associated with the incidence of MACE (all, p<0.05). Multivariate analysis indicated that in addition to chronic kidney disease, LVEF, Killip classification (all, p<0.05), the use of GTN showed an independent association with the incidence of MACE (HR 1.57; 95% CI 1.09–2.28; p=0.016).

**Table 2:** Univariate and multivariate analysis and inverse probability weighting on the predictor for MACE after PCI

	Univariate analysis			Multivariate analysis			Inverse probability weighting		
	HR	95% CI	P value	HR	95% CI	P value	HR	95% CI	P value
Age	1.02	1.01–1.04	**0.001**	1.00	0.99–1.02	0.558			
Male	0.76	0.54–1.07	0.117						
BMI	0.97	0.92–1.02	0.263						
Hypertension	1.08	0.74–1.60	0.680						
Dyslipidaemia	0.61	0.44–0.84	**0.003**	0.83	0.57–1.20	0.319			
Diabetes mellitus	1.21	0.86–1.70	0.274						
Chronic kidney disease	2.68	1.89–3.79	**<0.001**	1.61	1.07–2.41	**0.022**			
LVEF	4.53	3.18–6.47	**<0.001**	2.60	1.77–3.81	**<0.001**			
Killip classification	2.14	1.89–2.43	**<0.001**	1.75	1.50–2.04	**<0.001**			
STEMI	0.81	0.56–1.19	0.286						
Prior stroke	1.41	0.86–2.30	0.176						
GTN use before PCI	1.46	1.05–2.04	**0.026**	1.57	1.09–2.28	**0.016**	1.51	1.04–2.19	**0.028**

Bold values indicate statistical significance at the p <0.05 level.

Age=1-year increase; BMI categories=18.5−26, ≤18.5 or > 26; chronic kidney disease=eGFR≥60 mL/min per 1.73 m^2^, eGFR<60 mL/min per 1.73 m^2^; Killip classification=1, 2, 3, 4; LVEF≥50%, <50%.

BMI, body mass index; eGFR, estimated glomerular filtration rate; GTN, nitroglycerin; LVEF, left ventricular ejection fraction; MACE, major adverse cardiovascular events; MI, myocardial infarction; PCI, percutaneous coronary intervention; STEMI, ST-elevation myocardial infarction.

**Figure 2:**
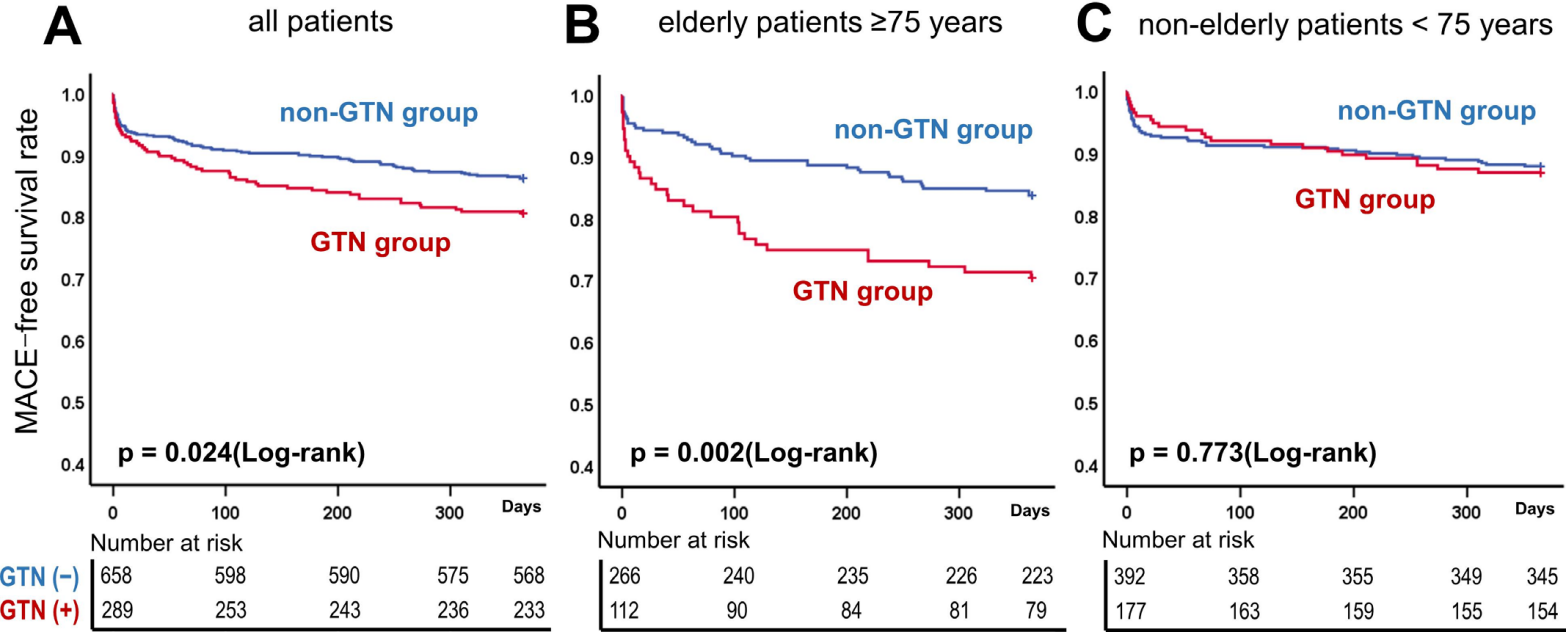
Comparison of the incidence of MACE using Kaplan-Meier curves between the GTN and non-GTN groups in all patients, elderly and non-elderly patients with ACS. (A) patients of all ages, (B) elderly patients (≥75 years), (C) non-elderly patients (<75 years). ACS, acute coronary syndrome; GTN, nitroglycerin; MACE, major adverse cardiac event.

As shown in figure 2A, Kaplan-Meier curves in all patients revealed that the incidence of MACE was significantly higher in the GTN group than in the non-GTN group (p=0.024). Next, the difference in MACE incidence between the GTN and non-GTN groups was analysed separately for elderly (age≥75 years) and non-elderly patients (age<75 years) (p interaction: 0.07); the significantly higher incidence of MACE in the GTN group than the non-GTN group was found only in elderly patients (p=0.002) (figure 2B) and not in non-elderly patients (p=0.773) (figure 2C).

Pre-PCI baseline characteristics of the elderly patients indicated that the GTN group had a significantly lower systolic blood pressure (132.0 (100.7–143.0) vs 134.0 (113.0–157.0) mm Hg, p=0.007 and higher prevalence of dyslipidaemia (76.7% vs 60.1%, p=0.002) than the non-GTN group. In addition, the GTN group in elderly patients tended to receive aspirin before ACS (25.0% vs 16.5%, p=0.056) (online supplemental table S4). Multivariate analysis in elderly patients indicated that age, LVEF, Killip classification and the use of GTN showed an independent association with the incidence of MACE (HR 1.05; 95% CI 1.00–1.10; p=0.037, HR 2.49; 95% CI 1.46–4.23; p=0.001, HR 1.53; 95% CI 1.23–1.90; p<0.001, HR 1.84; 95% CI 1.11–3.04; p=0.017) (table 3). As a sensitivity analysis, IPW showed similar results with multivariate analysis (tables 2 and 3). Each variable was well balanced (all variables<0.10).

**Table 3:** Univariate and multivariate analysis and inverse probability weighting on the predictor for MACE after PCI in elderly patients (≥75 years) with ACS

	Univariate analysis			Multivariate analysis			Inverse probability weighting		
	HR	95% CI	P value	HR	95% CI	P value	HR	95% CI	P value
Age	1.06	1.02–1.11	**0.003**	1.05	1.00–1.10	**0.037**			
Male	1.24	0.79–1.95	0.356						
BMI	0.99	0.92–1.06	0.797						
Hypertension	0.84	0.49–1.46	0.543						
Dyslipidaemia	0.77	0.49–1.22	0.271						
Diabetes mellitus	1.30	0.80–2.13	0.289						
Chronic kidney disease	1.73	1.04–2.88	**0.035**	1.07	0.60–1.91	0.810			
LVEF	3.66	2.23–6.00	**<0.001**	2.49	1.46–4.23	**0.001**			
Killip classification	1.79	1.50–2.14	**<0.001**	1.53	1.23–1.90	**<0.001**			
STEMI	0.93	0.55–1.58	0.798						
Prior stroke	1.13	0.61–2.09	0.699						
GTN use before PCI	2.02	1.28–3.18	**0.002**	1.84	1.11–3.04	**0.017**	1.72	1.08–2.75	**0.024**

Bold values indicate statistical significance at the p < 0.05 level.

Age=1-year increase; BMI categories=18.5–26, ≤18.5 or >26; chronic kidney disease=eGFR≥60 mL/min per 1.73 m^2^, eGFR<60 mL/min per 1.73 m^2^; Killip classification=1, 2, 3, 4; LVEF≥50%, <50%.

ACS, acute coronary syndrome; BMI, body mass index; eGFR, estimated glomerular filtration rate; GTN, nitroglycerin; LVEF, left ventricular ejection fraction; MACE, major adverse cardiovascular event; MI, myocardial infarction; PCI, percutaneous coronary intervention; STEMI, ST-elevation myocardial infarction.

## Discussion

This study investigated the association between GTN use preceding PCI for ACS and the incidence of MACE. The noteworthy findings are as follows: (1) Overall, the GTN group had lower pre-PCI blood pressure and a significantly higher incidence of MACE at 1-year follow-up than the non-GTN group, although peak CK level and LVEF after PCI for ACS were similar in the two groups. In addition to LVEF, chronic kidney disease and Killip classification, GTN use showed an independent association with the incidence of MACE. (2) The GTN group had a significantly higher incidence of MACE than the non-GTN group in elderly patients (age ≥75 years); however, the incidence of MACE did not differ between the GTN and non-GTN groups in non-elderly patients (age <75 years). (3) In both the GTN and non-GTN groups, patients who developed MACE had lower pre-PCI blood pressure than those who did not develop MACE. Moreover, the GTN group showed less pre-PCI systolic blood pressure than the non-GTN group among elderly patients, but we did not find such a difference between the GTN and non-GTN groups in non-elderly patients.

The latest guidelines from the Japanese Circulation Society,^6^ European Society of Cardiology^7^ and American College of Cardiology/American Heart Association^8^ recommend the administration of nitrates in the primary management for ACS, except in cases with contraindications such as marked hypotension, bradycardia and complicated right ventricular infarction. However, as Ekmejian *et al* recently described,^17^ these guidelines rely on the results from the GISSI-3^13^ and ISIS-4 study^14^ conducted more than 30 years ago when the treatment of ACS and the demographics of patients with ACS were significantly different from the current situation. Moreover, the results of these studies did not fully support the strong recommendations for the use of GTN for ACS. The percentage of patients with ACS undergoing primary PCI followed by OMT has increased markedly over the past 30 years, and these multidisciplinary treatments have significantly suppressed the development and progression of heart failure and the recurrence of ischaemic events. Additionally, the recent promotion of timely reperfusion after the onset of ST-elevation myocardial infarction includes shortening the D2B time to less than 90 min.^18 19^ Our hospital data also indicated that the D2B time was 73 min, meeting the goal stated in the guidelines.^6^ All of these initiatives help preserve cardiac function after ACS, resulting in that the haemodynamically beneficial effect of GTN in the acute phase, might have become relatively limited and less apparent compared with the era without widespread PCI and OMT.

The concerns regarding the safety of GTN use for ACS might have been relatively increased because the number and proportion of elderly patients with ACS increased, and elderly patients are more prone to hypotension after GTN administration.^20^ This study also confirmed that the GTN group showed less pre-PCI systolic blood pressure than the non-GTN group among elderly patients. Elderly patients 75 or more years of age account for 30%–40% of all hospitalised patients with ACS, and the incidence of ACS-associated death is also mainly observed in this age group.^21–23^ Similarly, this study included approximately 40% of patients aged 75 or more years, and the trend of outcomes for the study population as a whole and those in this age group were nearly identical. These findings suggest that the incidence of clinical events occurs mainly in elderly patients, which is similar to previous reports. Hence, optimisation of primary management for elderly patients with ACS is required,^24^ and the pros and cons of GTN use should be debated and examined separately for elderly and non-elderly patients.

The in-hospital and 1-year mortality among patients with ACS can be effectively estimated and identified by the GRACE score,^25 26^ which consists of eight items, including age and systolic blood pressure. The results of our study indicated a higher incidence of MACE in the GTN rather than the non-GTN group among elderly patients but not among non-elderly patients. Although this study lacks the GRACE score, poor outcomes in the elderly GTN group might be explained partly by risk elevation owing to the accumulation of older age and less systolic blood pressure. Potential mechanisms by which GTN use influences outcomes in elderly patients might include the pronounced blood pressure-lowering effect of GTN via vasomotor instability specific to the elderly population. A future study on whether the risk stratification score incorporating ‘GTN use before primary PCI’ can be developed in patients with ACS may be required.

This study had several limitations. First, the study was a single-centre cohort. Since the PCI strategy directly influences the incidence of MACE, generalising these single-centre results beyond strategical differences requires multicentre validation. Second, the results do not allow us to simply conclude that GTN use preceding primary PCI causally decreases blood pressure on admission and worsens clinical outcomes. There might be potential undetermined cofounders that influenced the present results; therefore, prospective randomised interventional studies to test the efficacy of GTN on clinical outcomes in ACS patients are warranted. Third, this study failed to show data on the time from the onset of chest symptoms to the use of GTN. Although there was no difference in peak CK elevation or LVEF between the GTN and non-GTN groups, the treatment, including GTN administration itself, might have prolonged the time until primary PCI from symptom onset. Finally, patients with ACS presenting with severe symptoms of chest pain might be more likely to be treated with GTN, suggesting that GTN use may be a marker for more severe ischaemia or more compromised patients. The clinical data regarding coronary severity included in this study (eg, the prevalence of multivessel disease) were compared between the GTN and non-GTN groups, but none showed a clear difference. However, if a more detailed and quantitative coronary severity grading system, such as the SYNTAX score, could have been incorporated into this study, potential confounding factors affecting outcomes in the GTN and non-GTN groups might have been identified.

In conclusion, GTN use preceding primary PCI was associated with adverse clinical outcomes in elderly patients with ACS. Further studies are needed to re-evaluate the impact of GTN use on clinical outcomes in these patients.

## Data Availability

Data are available on reasonable request. Raw data were generated at University of Miyazaki. Derived data supporting the findings of this study are available from the corresponding author on request. The data are not publicly available due to privacy reasons.
