# Supplementary material for: Nitroglycerin use and adverse clinical outcomes in elderly patients with acute coronary syndrome
Source: Open Heart. 2024 Jan 12;11(1):e002494. doi: 10.1136/openhrt-2023-002494 (PMC10806511; doi:10.1136/openhrt-2023-002494)
Supplement: Supplementary data [file openhrt-2023-002494supp001.pdf]

Supplementary Files for the article entitled "**Nitroglycerin use and adverse clinical outcomes in elderly patients with acute coronary syndrome**" by **Komaki et al.**

TABLE OF CONTENTS

Supplementary Methods S1: Design and study population - geographic characteristics of hospital location..……………………………………………………………………… **p 2**
Supplementary Methods S2: Definitions of coronary risk factors…………..……....... **p 2**
Supplementary Methods S3: PCI procedures……………………….……….…......**p 3**
Supplementary Table S1: Demographics in patients with ACS with/without GTN use.... **p 8**
Supplementary Table S2: Characteristics of ACS and PCI procedures in the GTN and non-GTN groups…………………………………………………………….…….………**p 9**
Supplementary Table S3: Baseline characteristics of the GTN group with and without the incidence of MACE ……………………………………………………………………**p 11**
Supplementary Table S4: Demographics in elderly and non-elderly patients with ACS with/without GTN use…………………………………………………..………………… **p 13**

Komaki S, *et al. Open Heart* 2024; 11:e002494. doi: 10.1136/openhrt-2023-002494

Supplementary Methods

S1: Design and study population - geographic characteristics of hospital location

Miyazaki Prefectural Nobeoka Hospital is the only regional hospital that plays a central role in acute intensive care and is the only facility in Nobeoka City with a cardiac catheterization laboratory where percutaneous coronary intervention can be performed (1,2). The nearest facility with a catheter laboratory is approximately 60 km from our hospital in another prefecture. The second nearest facility with a catheter laboratory is approximately 90 km from our hospital in another city in Miyazaki prefecture. Therefore, it is highly unlikely that patients with acute coronary syndrome (ACS) in Nobeoka City were transported by ambulance to facilities other than ours. These features of our hospital allowed us to calculate the accurate clinical outcomes of patients hospitalized for ACS in Nobeoka City.

S2: Definitions of coronary risk factors

In accordance with the Japanese Society of Hypertension 2009 Guidelines, hypertension was defined as a systolic blood pressure of 140 mmHg or more, a diastolic blood pressure of 90 mmHg or more, or receiving pharmacological treatment (3). Moreover, in accordance with the Japanese Atherosclerosis Society 2012 Guidelines, dyslipidemia was defined as when total cholesterol was 220 mg/dL or more, low-density lipoprotein cholesterol was 140 mg/dL or more, high-density lipoprotein cholesterol was less than 40 mg/dL, triglycerides were 150 mg/dL or more, or patients were receiving pharmacological treatment (4). Diabetes mellitus

was defined when the fasting plasma glucose level was 126 mg/dL or more, the casual plasma glucose level was 200 mg/dL or more, the HbA1c was 6.5% or more, the 2-hour plasma glucose level after 75 g glucose loading was 200 mg/dL or more, or the patient was receiving oral hypoglycemic agents and insulin.

S3: PCI procedures

All patients received aspirin, a P2Y12 inhibitor (clopidogrel or prasugrel), and weight-adjusted intravenous heparin before primary percutaneous coronary intervention (PCI) (5-9). Patients had a bolus injection of intravenous unfractionated heparin at a dose of 70 to 100 units/kg and maintenance of activated coagulation time at 250 seconds or longer. Invasive coronary angiography was performed with standard techniques. At least two different views were obtained for each main vessel. The extent of stenosis of each lesion was evaluated and recorded based on the visual assessment. The interventional cardiologist judged the indication of revascularization for culprit lesions. In primary PCI, the efficacy of revascularization of the culprit artery was comprehensively evaluated for improvement in angiographic findings, symptom relief, hemodynamic and electrical stabilization, and resolution of ST-segment elevation by electrocardiogram (ECG). Door-to-balloon time was measured as an index of early reperfusion in primary PCI for acute coronary syndrome (ACS) and aimed for a door-to-balloon time shorter than 90 minutes in accordance with the guideline (10). Successful reperfusion was defined as an acquisition of TIMI 3 flow after PCI.

Komaki S, *et al. Open Heart* 2024; 11:e002494. doi: 10.1136/openhrt-2023-002494

All patients with ACS after PCI were immediately and comprehensively treated in the coronary care unit while monitoring their ECG and vital signs. Cardiac rehabilitation was performed at an individually appropriate timing in reference to peak creatinine kinase level or the presence/absence of complications after ACS in the coronary care unit (11).

References

1. Marume K, Nagatomo K, Yamamoto N, et al. Prognostic impact of the presence of on-duty cardiologist on patients with acute myocardial infarction admitted during off-hours. J Cardiol 2020;76:184-90.

2. Ogata S, Marume K, Nakai M, et al. Incidence rate of acute coronary syndrome including acute myocardial infarction, unstable angina, and sudden cardiac death in Nobeoka City for the Super-Aged Society of Japan. Circ J 2021;85:1722-30.

3. Ogihara T, Kikuchi K, Matsuoka H, et al. The Japanese Society of Hypertension Guidelines for the Management of Hypertension (JSH 2009). Hypertens Res 2009;32:3-107. Erratum in: Hypertens Res 2014;37:599. Erratum in: Hypertens Res 2009;32:318.

4. Teramoto T, Sasaki J, Ishibashi S, et al. Executive summary of the Japan Atherosclerosis Society (JAS) guidelines for the diagnosis and prevention of atherosclerotic cardiovascular diseases in Japan -2012 version. J Atheroscler Thromb 2013;20:517-23.

5. Jolly SS, Pogue J, Haladyn K, et al. Effects of aspirin dose on ischaemic events and bleeding after percutaneous coronary intervention: insights from the PCI-CURE study. Eur Heart J 2009;30:900-7.

6. CURRENT-OASIS 7 Investigators, Mehta SR, Bassand JP, et al. Dose comparisons of clopidogrel and aspirin in acute coronary syndromes. N Engl J Med 2010;363:930-42.

7. Sabatine MS, Cannon CP, Gibson CM, et al. Effect of clopidogrel pretreatment before

percutaneous coronary intervention in patients with ST-elevation myocardial infarction treated with fibrinolytics: the PCI-CLARITY study. JAMA 2005;294:1224-32.

8. Ozaki Y, Hara H, Onuma Y, et al. CVIT expert consensus document on primary percutaneous coronary intervention (PCI) for acute myocardial infarction (AMI) update 2022. Cardiovasc Interv Ther 2022;37:1-34.

9. TIMI Study Group. The Thrombolysis in Myocardial Infarction (TIMI) trial. Phase I findings. N Engl J Med 1985;312:932-6.

10. Kimura K, Kimura T, Ishihara M, et al. JCS 2018 guideline on diagnosis and treatment of acute coronary syndrome. Circ J 2019;83:1085-196.

11. Thomas RJ, Denna T. The role of cardiac rehabilitation following acute coronary syndromes. Curr Cardiol Rep 2014;16:534.

BMJ Publishing Group Limited (BMJ) disclaims all liability and responsibility arising from any reliance placed on this supplemental material which has been supplied by the author(s)

Table S1: Demographics in patients with ACS with and without GTN use

	Total (n = 947)	GTN group (n = 289)	Non-GTN group (n = 658)	p-value
Height (cm)	161.8 (154.0-168.0)	162.0(156.0-168.0)	161.2 (153.0-168.0)	0.334
Body weight (kg)	60.7 (52.9-69.5)	62.0 (54.0-70.2)	60.3 (52.0-68.5)	0.052
BMI (kg/m^2)	23.4 (21.4-26.0)	23.7 (21.4-26.3)	23.4 (21.4-25.8)	0.114
Past history				
PCI, n (%)	140 (14.7)	52 (17.9)	88 (13.3)	0.065
CABG, n (%)	6 (0.6)	3 (1.0)	3 (0.4)	0.298
MI, n (%)	93 (9.8)	34 (11.7)	59 (8.9)	0.183
Stroke, n (%)	87 (9.1)	29 (10.0)	58 (8.8)	0.549
Antihypertensive therapy before ACS	501 (52.9)	151 (52.2)	350 (53.1)	0.789
Calcium channel blocker, n (%)	381 (40.2)	117 (40.4)	264 (40.1)	0.916
ACEi/ARB, n (%)	298 (31.4)	84 (29.0)	214 (32.5)	0.291
β blocker, n (%)	61 (6.4)	18 (6.2)	43 (6.2)	0.860
diuretic, n (%)	52 (5.4)	18 (6.2)	34 (4.9)	0.509
Antithrombotic drug use before ACS				
Aspirin, n (%)	147 (15.5)	60 (20.7)	87 (12.7)	**0.003**
Clopidogrel, n (%)	27 (2.8)	5 (1.7)	22 (3.2)	0.170
Prasugrel, n (%)	2 (0.2)	1 (0.3)	1 (0.1)	0.549
Warfarin, n (%)	11 (1.1)	7 (2.4)	4 (0.5)	**0.016**
Direct oral anticoagulants, n (%)	13 (1.3)	7 (2.4)	6 (0.8)	0.066

Data are presented as medians (interquartile ranges) or numbers (percentages).

ACEi, angiotensin-converting enzyme inhibitor; ACS, acute coronary syndrome; ARB, angiotensin II receptor blocker; BMI, body mass index; CABG, coronary artery bypass graft; GTN, nitroglycerin; MI, myocardial infarction; PCI, percutaneous coronary intervention.

Komaki S, *et al. Open Heart* 2024; 11:e002494. doi: 10.1136/openhrt-2023-002494

Table S2: Characteristics of ACS and PCI procedures

in the GTN and non-GTN groups

	Total<br>(n = 947)	GTN group<br>(n = 289)	Non-GTN group<br>(n = 658)	p-value
PCI approach vessel, n (%)<br>Radial artery<br>Brachial artery<br>Femoral artery	 796 (84.0)<br>86 (9.0)<br>65 (6.8)	 251 (86.8)<br>23 (7.9)<br>15 (5.1)	 545 (82.8)<br>63 (9.5)<br>50 (7.5)	0.348
PCI lesion, n (%)<br>LMT<br>LAD<br>LCX<br>RCA<br>SVG	 36 (3.8)<br>403 (42.5)<br>137 (14.4)<br>370 (39.0)<br>1 (0.1)	 10 (3.4)<br>126 (43.5)<br>54 (18.6)<br>98 (33.9)<br>1 (0.3)	 26 (3.9)<br>277 (42.0)<br>83 (12.6)<br>272 (41.3)<br>0 (0.0)	0.108
Culprit lesion RCA, n (%)	370 (39.0)	98 (33.9)	272 (41.3)	**0.031**
Multivessel coronary artery disease, n (%)	388 (40.9)	122 (42.2)	266 (40.4)	0.606
Killip Classification, n (%)<br>1<br>2<br>3<br>4	 594 (62.7)<br>164 (17.3)<br>60 (6.3)<br>129 (13.6)	 183 (63.3)<br>45 (15.5)<br>22 (7.6)<br>39 (13.4)	 411 (62.4)<br>119 (18.0)<br>38 (5.7)<br>90 (13.6)	0.927
PCI procedure, n (%)<br>Drug-eluting stents<br>Bare metal stents<br>POBA, only<br>Aspiration, only	 805 (85.0)<br>74 (7.8)<br>53 (5.5)<br>15 (1.5)	 239 (82.6)<br>22 (7.6)<br>21 (7.2)<br>7 (2.4)	 566 (86.0)<br>52 (7.9)<br>32 (4.8)<br>8 (1.2)	0.241
Peak CK level, (units/L)	1762.0<br>(724.7-3458.5)	1740.0<br>(736.5-3512.0)	1824.0<br>(713.0-3459.0)	0.920
Door-to-balloon time, (min)	73.0 (58.0-102.0)	73.0 (58.0-95.0)	73.0 (58.0-106.0)	0.245
Use of imaging device, n (%)	934 (98.6)	283 (97.9)	651 (98.9)	0.218
Cardiac and respiratory assist devices, n (%)	164 (17.3)	41 (14.1)	123 (18.6)	0.092
IABP, n (%)	95 (10.0)	31 (10.7)	64 (9.7)	0.637
ECMO, n (%)	26 (2.7)	7 (2.4)	19 (2.8)	0.687
Temporary pacing, n (%)	87 (9.1)	17 (5.8)	70 (10.6)	**0.020**
NPPV or ventilator, n (%)	80 (8.4)	18 (6.2)	62 (9.4)	0.104
PCI Failure, n (%)	5 (0.5)	1 (0.3)	4 (0.6)	0.609
Antithrombotic medication at discharge				
Aspirin, n (%)	937 (98.9)	284 (98.2)	653 (99.2)	0.179
Clopidogrel, n (%)	369 (38.9)	106 (36.6)	263 (39.9)	0.428
Prasugrel, n (%)	565 (59.6)	176 (60.8)	389 (59.1)	0.563
Warfarin, n (%)	22 (2.3)	10 (3.4)	12 (1.8)	0.283
Direct oral anticoagulants, n (%)	59 (6.2)	23 (7.9)	36 (5.4)	0.145

Data are presented as medians (interquartile ranges) or numbers (percentages).

Komaki S, *et al. Open Heart* 2024; 11:e002494. doi: 10.1136/openhrt-2023-002494

CK, creatine kinase; ECMO, extracorporeal membrane oxygenation; GTN, nitroglycerin; IABP, intra-aortic balloon pumping; LAD, left anterior descending artery; LCX, left circumflex artery; LMT, left main coronary trunk; NPPV, non-invasive positive airway pressure ventilation; PCI, percutaneous coronary intervention; POBA, percutaneous old balloon angioplasty; RCA, right coronary artery; STEMI, ST-elevation myocardial infarction; SVG, saphenous vein graft.

Komaki S, *et al. Open Heart* 2024; 11:e002494. doi: 10.1136/openhrt-2023-002494

Table S3: Baseline characteristics of the GTN group with and without the incidence of MACE

	GTN group with MACE (n = 56)	GTN group without MACE (n = 233)	p-value
Age (years)	77.0 (68.2-86.0)	68.0 (60.0-78.0)	**< 0.001**
Elderly (≥75 years), n (%)	33 (58.9)	79 (33.9)	**< 0.001**
Male, n (%)	38 (67.8)	177 (75.9)	0.212
Height (cm)	159.0 (153.5-165.0)	163.0 (156.9-168.0)	**0.030**
Body weight (kg)	56.6 (49.2-68.0)	62.6 (55.3-71.0)	**0.015**
BMI (kg/m^2)	23.0 (21.2-25.8)	23.9 (21.4-26.4)	0.240
Waist circumference (cm)	90.0 (82.0-97.0)	87.5 (82.0-94.0)	0.446
Systolic blood pressure (mmHg)	130.0 (95.0-141.0)	132.0 (113.0-146.0)	**0.011**
Diastolic blood pressure (mmHg)	76.5 (57.7-91.2)	84.0 (70.0-95.0)	**0.011**
Previous/current smoking, n (%)	22 (39.2)	117 (50.2)	0.142
Hypertension, n (%)	47 (83.9)	192 (82.4)	0.786
Dyslipidemia, n (%)	40 (71.4)	182 (78.1)	0.287
Diabetes mellitus, n (%)	25 (44.6)	75 (32.1)	0.079
Metabolic syndrome, n (%)	13 (23.2)	99 (42.4)	**0.005**
Hemodialysis, n (%)	4 (7.1)	6 (2.5)	0.093
TG (mg/L)	93.0 (72.5-132.0)	107.0 (72.0-154.0)	0.429
HDL-C (mg/L)	42.5 (38.2-52.7)	50.0 (42.5-59.0)	**0.001**
LDL-C (mg/L)	110.0 (87.0-140.2)	124.0 (99.5-155.0)	**0.034**
HbA1c (%)	6.30 (5.60-7.55)	5.90 (5.60-6.50)	0.068
eGFR (mL/min per 1.73 m^2)	45.6 (29.4-63.5)	63.8 (52.2-75.2)	**<0.001**
LVEF (%)	45.8 (35.5-52.7)	58.0 (53.0-62.8)	**< 0.001**
Past history			
PCI, n (%)	13 (23.2)	39 (16.7)	0.257
CABG, n (%)	1 (1.7)	2 (0.8)	0.539
MI, n (%)	13 (23.2)	21 (9.0)	**0.003**
Stroke, n (%)	10 (17.8)	19 (8.1)	**0.030**
Antihypertensive therapy before ACS, n (%)	27 (48.2)	124 (53.2)	0.501
Calcium channel blocker, n (%)	20 (35.7)	97 (41.6)	0.418
ACEi/ARB, n (%)	13 (23.2)	71 (30.4)	0.283
β blocker, n (%)	7 (12.5)	11 (4.7)	**0.031**
Diuretic, n (%)	7 (12.5)	11 (4.7)	**0.031**
Antithrombotic drug use before ACS			
Aspirin, n (%)	14 (25.0)	46 (19.7)	0.384
Clopidogrel, n (%)	0 (0)	5 (2.1)	0.269
Prasugrel, n (%)	0 (0)	1 (0.4)	0.623
Warfarin, n (%)	1 (1.7)	6 (2.5)	0.730
Direct oral anticoagulants, n (%)	3 (5.3)	4 (1.7)	0.112

Komaki S, *et al. Open Heart* 2024; 11:e002494. doi: 10.1136/openhrt-2023-002494

STEMI, n (%)	45 (80.3)	181 (77.6)	0.663

Data are presented as medians (interquartile ranges) or numbers (percentages).

ACEi, angiotensin-converting enzyme inhibitor; ACS, acute coronary syndrome; ARB, angiotensin II receptor blocker; BMI, body mass index; CABG, coronary artery bypass graft; eGFR, estimated glomerular filtration rate; GTN, nitroglycerin; HDL-C, high-density lipoprotein cholesterol; LDL-C, low-density lipoprotein cholesterol; LVEF, left-ventricular ejection fraction; MACE, major adverse cardiovascular events; MI, myocardial infarction; PCI, percutaneous coronary intervention; STEMI, ST-elevation myocardial infarction; TG, triglyceride.

Komaki S, *et al. Open Heart* 2024; 11:e002494. doi: 10.1136/openhrt-2023-002494

Table S4: Baseline characteristics in elderly and non-elderly patients with ACS with/without GTN use

	Elderly (≥ 75 years) (n = 378)			Non-elderly (< 75 years) (n = 569)		
	GTN group (n = 112)	Non-GTN group (n = 266)	p-value	GTN group (n = 177)	Non-GTN group (n = 392)	p-value
Age (years)	82.0 (78.2-86.0)	82.0 (78.0-86.0)	0.951	64.0 (56.0-69.0)	64.0 (57.2-70.0)	0.296
Male, n (%)	64 (57.1)	136 (51.1)	0.285	151 (85.3)	327 (83.4)	0.569
Height (cm)	155.0 (146.8-162.0)	154.0 (146.8-161.1)	0.871	165.5 (160.0-170.0)	165.0 (159.7-169.0)	0.303
Body weight (kg)	55.0 (46.1-61.3)	54.0 (45.9-60.9)	0.464	66.3 (59.0-74.3)	64.6 (57.6-72.7)	0.096
BMI (kg/m^2)	22.4 (20.3-26.0)	22.8 (20.2-24.8)	0.715	24.5 (22.3-26.4)	23.8 (21.8-26.2)	0.135
Waist circumference (cm)	84.7 (78.6-91.0)	85.0 (80.0-93.0)	0.632	89.5 (83.0-96.0)	87.5 (82.0-93.0)	**0.034**
Systolic blood pressure (mmHg)	132.0 (100.0-143.0)	134.0 (113.7-157.0)	**0.007**	132.0 (119.0-144.0)	133.0 (112.0-157.0)	0.511
Diastolic blood pressure (mmHg)	74.0 (64.0-87.0)	75.0 (64.0-86.2)	0.868	84.0 (69.0-97.0)	87.0 (73.0-98.0)	0.380
Previous/current smoking, n (%)	28 (25.0)	74 (27.8)	0.573	111 (62.7)	252 (64.2)	0.718
Hypertension, n (%)	96 (85.7)	210 (78.9)	0.126	143 (80.7)	268 (68.3)	**0.002**
Dyslipidemia, n (%)	86 (76.7)	160 (60.1)	**0.002**	136 (76.8)	275 (70.1)	0.099
Diabetes mellitus, n (%)	29 (25.8)	69 (25.9)	0.992	71 (40.1)	131 (33.4)	0.122
Metabolic syndrome, n (%)	24 (21.4)	61 (22.9)	0.802	88 (49.7)	157 (40.0)	0.070
Hemodialysis, n (%)	2 (1.7)	6 (2.2)	0.772	8 (4.5)	13 (3.3)	0.481
TG (mg/L)	89.0 (69.0-128.0)	84.0 (62.0-123.5)	0.220	117.0 (84.0-169.7)	126.0 (85.0-207.0)	0.053
HDL-C (mg/L)	49.0 (41.2-62.5)	49.0 (41.0-60.7)	0.643	48.0 (41.0-58.0)	47.0 (39.0-55.5)	0.190
LDL-C (mg/L)	117.5 (94.2-141.0)	112.0 (89.0-140.0)	0.389	127.0 (99.0-160.0)	129.0 (102.0-159.0)	0.892
HbA1c (%)	5.80 (5.50-6.40)	5.80 (5.40-6.20)	0.514	6.00 (5.63-7.07)	5.90 (5.53-6.78)	0.109
eGFR (mL/min per 1.73 m^2)	52.0 (36.2-63.6)	52.7 (40.2-64.4)	0.224	66.7 (55.0-81.0)	65.0 (54.0-77.6)	0.231
LVEF (%)	54.5 (45.8-61.4)	55.7 (47.4-61.5)	0.579	57.4 (51.7-62.6)	57.4 (50.0-62.7)	0.661
Past history						
PCI, n (%)	21 (18.7)	39 (14.6)	0.321	31 (17.5)	49 (12.5)	0.111
CABG, n (%)	2 (1.7)	2 (0.7)	0.370	1 (0.5)	1 (0.2)	0.563
MI, n (%)	15 (13.3)	26 (9.7)	0.302	19 (10.7)	33 (8.4)	0.375
Stroke, n (%)	18 (16.0)	36 (13.5)	0.520	11 (6.2)	22 (5.6)	0.776
Antihypertensive therapy before ACS, n (%)	71 (63.3)	182 (68.4)	0.343	80 (45.1)	168 (42.8)	0.602
Calcium channel blocker, n (%)	55 (49.1)	137 (51.5)	0.670	62 (35.0)	127 (32.3)	0.537
ACEi/ARB, n (%)	36 (32.1)	107 (40.2)	0.139	48 (27.1)	107 (27.2)	0.965
β blocker, n (%)	8 (7.1)	20 (7.5)	0.899	10 (5.6)	23 (5.8)	0.918
Diuretic, n (%)	12 (10.7)	19 (7.1)	0.248	6 (3.3)	15 (3.8)	0.798
Antithrombotic drug use before ACS						
Aspirin, n (%)	28 (25.0)	44 (16.5)	0.056	32 (18.0)	43 (10.9)	**0.020**
Clopidogrel, n (%)	3 (2.6)	9 (3.3)	0.721	2 (1.1)	13 (3.3)	0.132
Prasugrel, n (%)	0 (0)	1 (0.3)	0.516	1 (0.5)	0 (0)	0.136
Warfarin, n (%)	2 (1.7)	4 (1.5)	0.841	5 (2.8)	0 (0)	**<0.001**
Direct oral anticoagulants, n (%)	5 (4.4)	5 (1.8)	0.153	2 (1.1)	1 (0.2)	0.182

Komaki S, *et al. Open Heart* 2024; 11:e002494. doi: 10.1136/openhrt-2023-002494

STEMI, n (%)	88 (78.5)	206 (77.4)	0.810	138 (77.9)	316 (80.6)	0.467

Data are presented as medians (interquartile ranges) or numbers (percentages).

ACEi, angiotensin-converting enzyme inhibitor; ACS, acute coronary syndrome; ARB, angiotensin II receptor blocker; BMI, body mass index; CABG, coronary artery bypass graft; eGFR, estimated glomerular filtration rate; GTN, nitroglycerin; HDL-C, high-density lipoprotein cholesterol; LDL-C, low-density lipoprotein cholesterol; LVEF, left-ventricular ejection fraction; MACE, main adverse cardiac events; MI, myocardial infarction; PCI, percutaneous coronary intervention; STEMI, ST-elevation myocardial infarction; TG, triglyceride.

Komaki S, *et al. Open Heart* 2024; 11:e002494. doi: 10.1136/openhrt-2023-002494
